# Circulating Tumour Cells as Prognostic Biomarkers in Colorectal Cancer: A Systematic Review

**DOI:** 10.3390/ijms22083437

**Published:** 2021-04-08

**Authors:** Léa Veyrune, David N. Naumann, Niki Christou

**Affiliations:** 1 Department of Cytogenetics, Medical Genetics and Reproductive Biology, University Hospital Limoges, Avenue Martin Luther King, CEDEX 87042 Limoges, France; lea.veyrune@gmail.com; 2 General Surgery Department, University Hospitals Birmingham NHS Foundation Trust, Mindelsohn Way, Edgbaston, Birmingham B15 2GW, UK; david.naumann@nhs.net; 3 Digestive Surgery Departement, University Hospital Limoges, Avenue Martin Luther King, CEDEX 87042 Limoges, France; 4 EA3842 CAPTuR Laboratory “Cell Activation Control, Tumour Progression and Therapeutic Resistance”, Faculty of Medicine, 2 rue du Docteur Marcland, 87025 Limoges, France

**Keywords:** circulating tumour cells, colorectal cancer, prognosis biomarker

## Abstract

Despite therapeutic advances, colorectal cancer (CRC) is still one of the deadliest cancers, partly due to local recurrence and metastatic disease. Tumour cells that spread by gaining access to peripheral blood are called circulating tumour cells (CTCs). These may be present before there are any clinical signs, but can be detected within blood samples. CTCs from patients with CRC may be isolated in a laboratory for characterization and multiple analyses. In this review, we focus on the prognostic potential of CTCs detection, by evaluating the reported progress and applications of such analyses. Our search found 77 relevant studies that reported CTC detection in CRC. Both cell count and features were reported as promising prognosis biomarkers. Since CTCs are rare and can lose their differentiation, new tools are being developed to improve detection. CTCs may have potential as prognostic biomarkers for CRC in terms of survival prediction, anticipating chemotherapy resistance, and surgical planning. CTCs are not yet used in clinical practice, and further investigations are required in order to better frame their practical value.

## 1. Introduction

Colorectal cancer (CRC) is the third most common cause of cancer-related death worldwide [1]. This high mortality may be explained by the relatively high incidence and high rates of local regional and metastatic disease in CRC. The incidence is increasing in the context of organised screening programmes and an ageing population. The preponderance of advanced and metastatic disease is still a reality despite research advances in treatment. Quality of life is a vital aspect of our care for patients with CRC, and individualised treatments may help to limit side effects. Since prognostic biomarkers may allow for more tailored treatments for our patients, circulating tumour cells (CTCs) have potential to be helpful in this context.

The aim of this systematic review is to examine how CTCs in patients with CRC can be used as prognostic biomarkers. We will summarise the progress reported in the literature so far, and will discuss future research.

## 2. Circulating Tumour Cells (CTCs): Isolation Enrichment and Detection

CTCs are epithelial cancer cells from the primary tumour or metastases that gained access to the circulatory system, and are detectable in sampled peripheral blood. They are believed to be directly involved in the biology of the metastatic process [2]. From this perspective, CTCs may then be defined as surrogate tumour material.

Different techniques have been developed to isolate CTCs from the bloodstream, based on biophysical methods (deformability, size, density, and surface charge), and/or immunoaffinity status.

CellSearch is the only FDA-approved (Food Drug Administration) technology for CTC isolation. It identifies Epithelial Cell Adhesion Molecule (EpCAM) expressing cells thanks to antibody-labelled magnetic nanoparticles and the use of fluorescent microscopy to detect cytokeratin()+ CK(+), DAPI(+), and CD45(−) cells [EpCAM(+)/CK(+)/CD45(−)/DAPI(+)] [3].

Once CTCs are isolated, they are available for multiple analyses such as genetic, epigenetic, transcriptomic, proteomic, and identification of surface cell markers or living cell properties. All these analyses have the potential to be used in the clinical setting for either diagnosis, prognosis, prediction of recurrences, or to adapt therapeutics for different types of solid cancers [4].

## 3. Review Methodology

We searched for published studies that reported the use of CTCs as a prognostic marker for CRC patients. Our search was performed with PubMed from 1975 to 1 February, 2021. As search terms, we used “colorectal cancer (CRC)”, “circulating tumour cells (CTC)”, and “prognosis biomarker”. All articles in English or French reporting information about CTCs, CRC and prognosis were included in our initial search. We excluded all articles where the study population was not patients with CRC. Case reports were excluded. Any articles about CTCs that did not integrate prognostic factors were also excluded.

Articles were eligible for inclusion if they reported either quantitative data (presence/absence or number of CTCs) or qualitative data (specific CTC elements).

**Figure 1:**
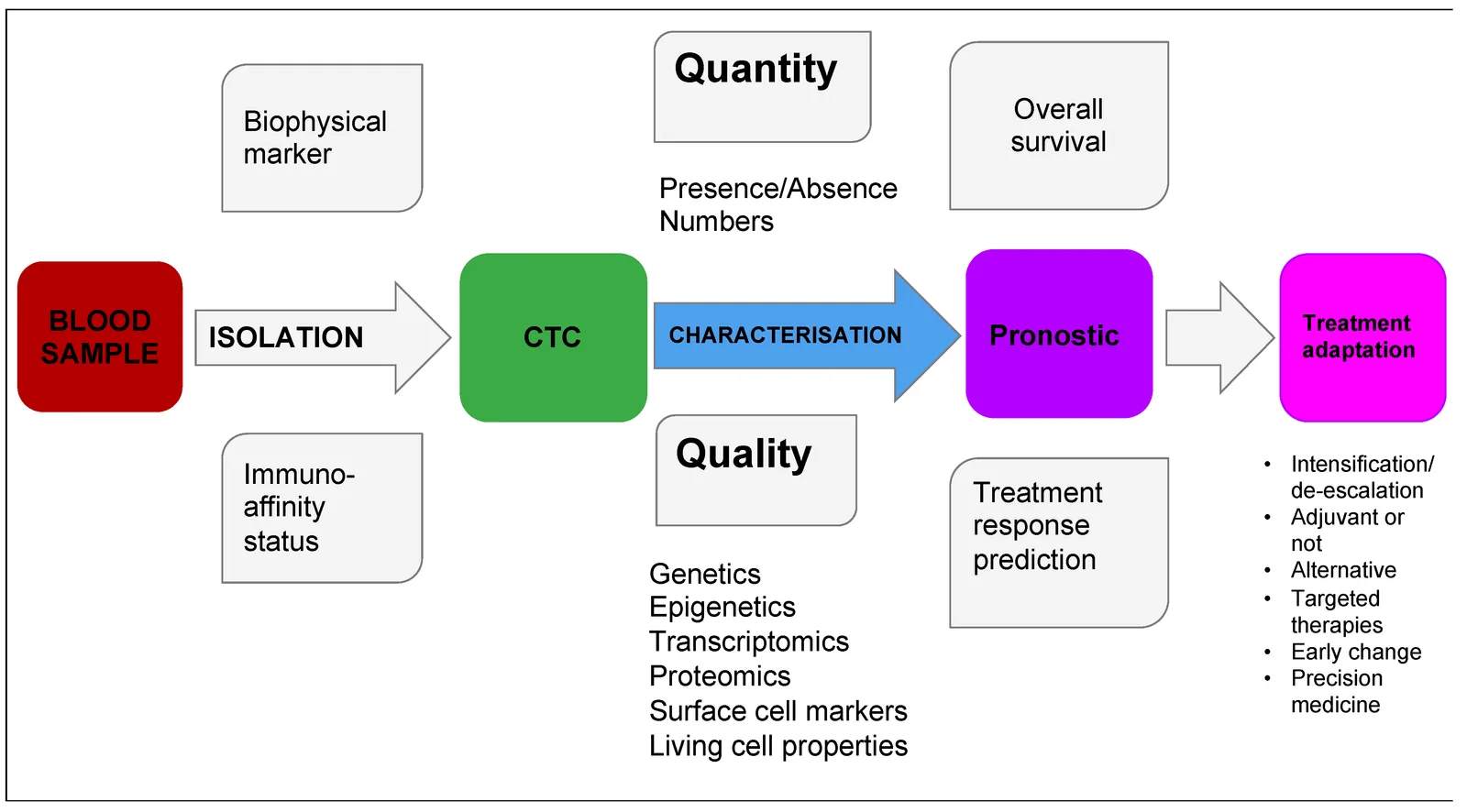
Colorectal cancer (CRC) circulating tumour cells (CTCs), from blood sample to clinical application.

For the definition of “prognosis”, we included articles that discussed overall survival regardless of the treatment, and those that discussed the sensitivity or resistance to a specific treatment. We were also interested in discussions regarding treatment modulation, such as adding or cancelling treatment, drug intensification or de-escalation, possibility of targeted therapy, alternative treatment including early change or interruption of active care (Figure 1). These were of interest because they facilitate precision medicine that enables us to give the best care with the least side effect as possible.

## 4. Results

**Figure 2:**
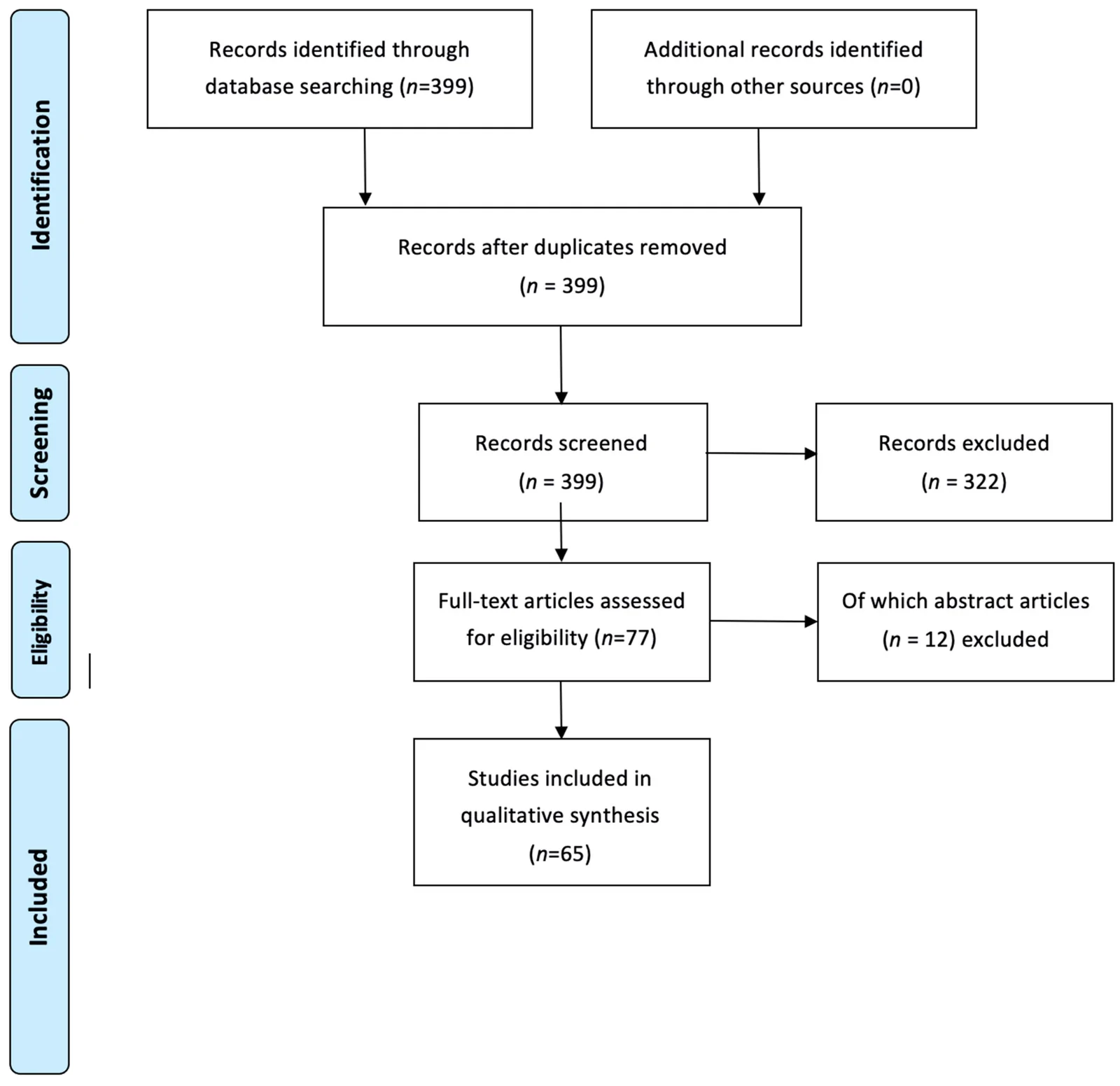
Flowchart.

**Figure 3:**
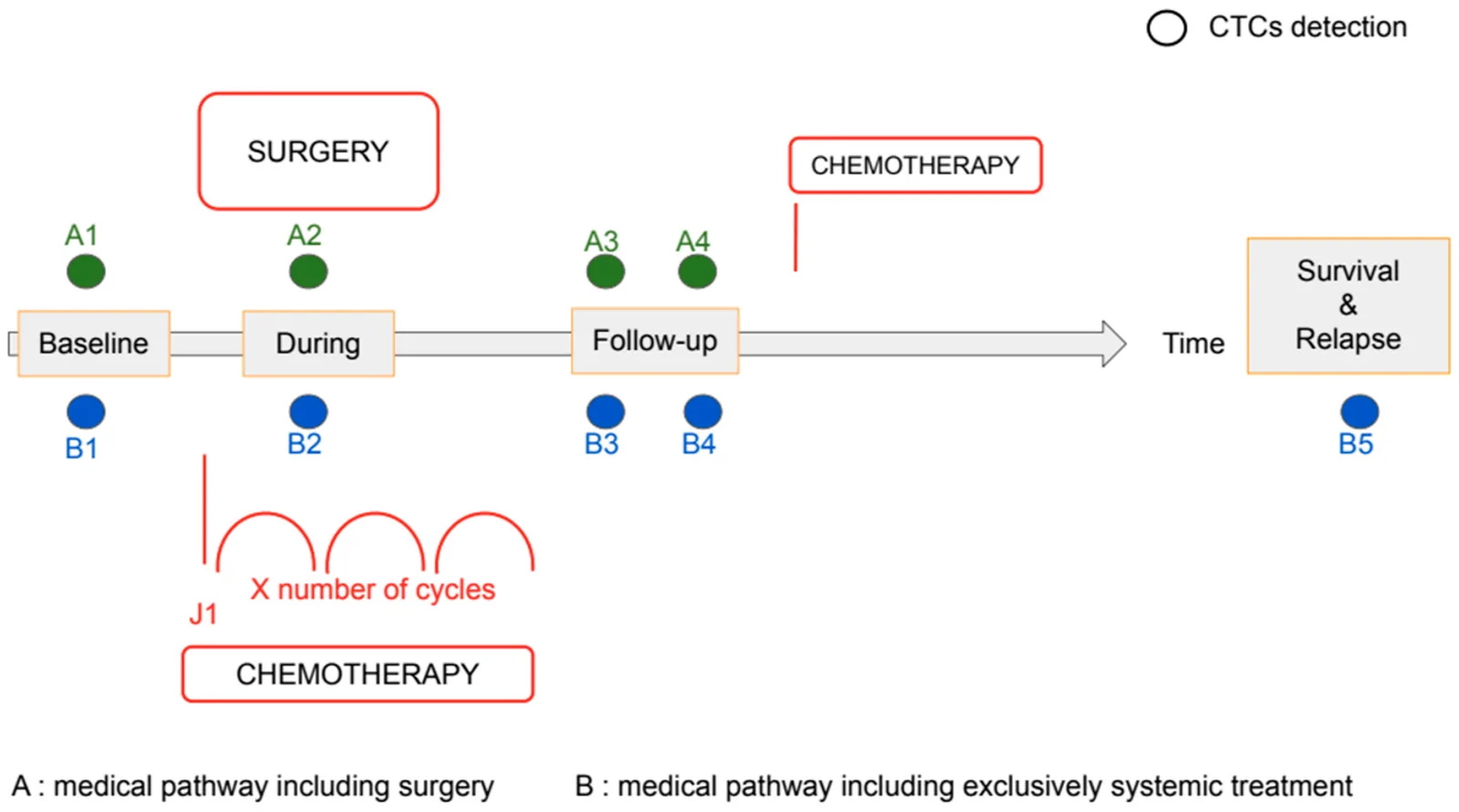
Time point for CTCs detection for colon cancer without neo-adjuvant treatment.

We found 399 articles in our initial search. After excluding reports (Table 1) not meeting the selection criteria (Table 1), there were 65 relevant studies (Figure 2). We classified studies according to type of marker used (quantitative, qualitative or both), techniques (Table 2), and their CTC detection time points (Figure 3). They are summarised in Table 3, Table 4 and Table 5.

### 4.1. Types of Markers

The majority of articles use CTCs as a quantitative prognostic biomarker (Table 2). This is likely to be due to the only FDA-approved method (CellSearch) being a CTC count method. CellSearch, an EpCAM based technique, was used in 14 of the 77 included studies.

EpCAM is part of the cell adhesion molecule (CAM) family. It was first identified in colon cancer in 1979 [70]. Epithelial Cell Adhesion Molecule (EpCAM) is expressed by normal epithelial cells and is highly expressed in a majority (70%) of primary tumour.

Concerning colon adenocarcinomas, EpCAM is overexpressed in 81% [71] and it is known to promote tumour expansion. EpCAM is involved in numerous independent pathways [72]. It plays a part in the activation of the Wnt signalling pathway that is known for its role in carcinogenesis. On one hand, EpCAM activates this pathway through its interaction and stabilization of Lrp6. On the other hand, it downregulates the expression of some this pathway inhibitors (fgf3). EpCAM is also known to promote oncogenesis through its interaction with β -catenin, by activating cell proliferation via proto-oncogenes, such as *c-Myc* or *Cyclin A* [73].

Thus, EpCAM is often use to characterise CTCs. However, during metastatic spread, epithelial–mesenchymal transition (EMT) occurs and tumour cells lose their epithelial marker, one of them being EpCAM. This cancer expansion modality highlights a clear limit of anti-EpCAM based detection system [74].

In addition to this loss of specific markers, CTCs are also difficult to separate because of their low concentration in the blood. To tackle these difficulties, newer strategies have been proposed, such as the using blood samples close to the tumour, such as mesenteric and portal vein blood samples, and detection methods such as specific gene reverse transcriptase quantitative polymerase chain reaction (RT-qPCR). This quantitative RNA-based method for CTC detection is more sensitive but has limitations. Target genes need to be carefully selected as they can be weakly expressed in normal blood cells leading to false-positives. Moreover if chosen target genes are expressed differently from one CTC to another, this may lead to an inaccurate tumour cell count [75].

After total mononuclear cell RNA extraction, RT-PCR is conducted on mRNA that are supposed to be specific to CTCs. More and more genes are being explored, focusing on different CTCs characteristics, such as epithelial (CEA, CK20, and CK19) and stem cells–like potential.

### 4.2. Prognostic Thresholds

For studies that utilised the CellSearch system, there were threshold changes according to CRC stage. For metastatic CRC, CTC count above or equal to 3 CTC per 7.5 mL of blood was used by Matsusaka et al., 2011 [5], Aggarwal et al., 2013 [7], and Sastre et al., 2013 [9] as a positive marker to determine “high CTC” patients. These studies all reported a significant correlation between baseline “high CTC” status and reduced survival. Camera et al., 2020 [17], similarly reported a prognostic difference in the presence of disease refractory to standard treatment or unresectable CRC. Coumans et al., 2012 [6], reported that survival for patients with metastatic CRC patients with CTCs is reduced by 6.6 months for each 10-fold CTC increase.

Krebs et al., 2015 [11] and Aranda et al., 2020 [16] showed benefits of escalating therapy from a three-drug regimen to a four-drug chemotherapy regimen for patients above their CTC prognostic threshold.

For patients undergoing surgery, CTC count above or equal to 1 CTC per 7.5 mL of blood was used by Bork et al., 2015 [10] and van Dalum et al., 2015 [13] to determine survival. These finding suggest a reduced surgical utility for patients above the prognostic threshold.

### 4.3. Techniques

There were multiple quantitative techniques other than CellSearch used in the included studies. The epithelial markers CK20 and CEA were the most commonly used among included studies. Taniguchi et al., 2000 [76] used CEA whereas Katsumata et al., 2006 [24], and Hinz et al., 2017 [44], used CK2O, and reported a significant association between CTC at time of surgery and survival. Allen-Mersh et al., 2007 [25] and Ito et al., 2002 [21] used either one or both of these markers to investigate the correlation between CTC detection after surgery and survival. For rectal cancer patients, CK20 was chosen by Hinz et al., 2015 [39], to predict non-response among rectal cancer undergoing neo-adjuvant chemoradiation.

Other newer techniques are being developed such as combined markers detection [31] or new molecule presence, like survivin [29].

### 4.4. Qualitative Data from CTCs

There was some overlap in the reporting of quantitative and qualitative markers. When CTC are detected without being destroyed, many complementary qualitative analyses are available. Study investigators have focused on markers of the aggressiveness of disease, expressed as either proteins (surface cell marker) or genes.

New marker development is focusing on discovering specific CTC subpopulations that may have particular effects on prognosis. Zhao et al., 2017 [67] combined CTC count and EMT state profile to predict disease aggressiveness. Tseng et al., 2015 [58] correlated the presence of CD133+ CD44+ CD54+ CTC subpopulation with lower survival in metastatic patients that did not undergo metastasis resection. Abdallah et al., 2016 [59] used MRP1 positive CTC as a prognosis marker in patient that were going to initiate a new chemotherapy agent.

Since markers are not always easy to determine [64], some clinicians try to associate their detection with other techniques. One of them is to collect blood closer from the tumour. Tseng et al., 2015 [58], used CTC analysis from mesenteric venous blood collected while the patient was on the operating table.

## 5. Discussion

### 5.1. Main Findings

The main finding from our search of the literature regarding the prognostic utility of CTCs for patients with CRC is that there are multiple quantitative and qualitative tests that have been used, with some success, in predicting overall survival and success of treatment, such as surgery and chemotherapy.

The presence of CTCs is a key risk factor for disease progression and severity. In breast cancer, they are considered as efficient prognostic biomarkers and included in the 7th AJCC Cancer Staging Manual (new category M0(i) introduced, and defined “by the presence of circulating or disseminated tumour cells not exceeding 0.2 mm detectable in bone marrow, circulating blood or other non-regional tissues of non-metastatic patients”. For patients with CRC, this type of staging is not yet undertaken. Indeed, there is ongoing debate regarding the utility of CTC detection due to the variations in techniques and conflicting results [77,78]. Our review confirmed that there are a range of heterogeneous techniques and results.

Most of our selected studies integrate metastatic patients (46/77). Additional prospective studies that investigate early CRC are needed in order to determine whether CTCs have any prognostic utility in this context. Rectal cancer was the subject for only 3 of the 77 included studies, and requires further evaluation in prognostic studies.

Isolation and examination of CTCs allows different analyses focusing on a variety of cell elements. The studies included in our review show that whatever quantitative or qualitative techniques (or both) are used, CTCs have potential as biomarkers to predict differences in outcomes such as survival and disease prognosis. To improve CTCs detection, new biomarkers are being studied and selected in order to help distinguish CTCs from other cells such as hematopoietic cells.

### 5.2. Detecting Circulating Tumour Cells

Since CTCs are epithelial cells that are found in the circulation, one option to detect their presence would be to use epithelial markers, such as CEA and cytokeratins. CEA is part of the immunoglobulin superfamily and is the product of the CEACAM5 gene [79], and is involved in cellular adhesion. It is commonly found on the surface of small and large bowels, rectum, pancreas, lung, and kidney cells. Being at a low rate for healthy people, it is already being used in routine clinical practice as a biomarker for CRC. However, its specificity is not the best as it can be increased in heavy smokers, inflammatory bowel disease, chronic obstructive pulmonary disease, pancreatitis or other adenocarcinomas than colorectal (such as ovaries, lung, kidney). On the RNA side, CEA mRNA can be detected in almost all epithelial cells, including CTCs, and is not found in non-epithelial cells [80]. Cytokeratins are part of the intermediate filaments of epithelial cells cytoskeleton. They are expressed in a tissue-specific manner. CK20 expression is limited to the gastric and intestinal epithelium, urothelium, and Merkel cells, and from malignancies that originate from these sites [30,81,82]. CK20 is not expressed in hematopoietic cells [83]. The main limitation of these biomarkers is that cancer cells undergo epithelial–mesenchymal transition (EMT) in the circulation, which can cause down regulation of epithelial markers. Therefore, these markers will only partially detect CTCs.

Yokobori et al., 2013 [37] chose to detect CTCs by using a marker that is not lost during the EMT process: plastin3 (PLS3). It codes for a ubiquitous protein that inhibits depolymerization of actin fibres. PLS3 is actually an EMT inducer and is therefore over-expressed by CTCs [84]. While working on EMT, Armstrong et al., 2011 [85] found that CTCs expressed both epithelial, and stem cell markers. Some of the included studies utilised stemness markers such as survivin, CD44 variants 6 and 9, and CD133. A good strategy seems to combine both categories of markers (epithelial and stemness) [42]. Survivin is an inhibitor of apoptosis and is highly conserved in CTCs cells. By limiting this programmed cell death, tumour cells develop aggressiveness [86]. Survivin is found in many cancer tissues, including CRC [29] and is not expressed in normal ones [87].

CD44 is a gene involved in adhesion cells and growth and invasion in tumour cells. CD44 is expressed by metastasis initiating cells [88] also known as cancer stem cells in CRC, but its exact function in these particular cells is still unknown, especially the exon that plays the central role [89]. CD133 (also known as prominin-1), is a transmembrane protein, initially described on the surface of hematopoietic stem cells; it is now known as a stemness marker for normal and cancer cells. It is not specific for CRC [90], but is considered as a key marker of tumoural stem cells in CRC [31]. Metastatic colon tissues are formed of CD133 (+) and (−) cells that can both initiate tumour cells. Since CD133 is also known to be expressed by endothelial cells, its expression by RT-PCR might be caused by bone marrow-derived circulating endothelial cells [35].

Other biomarkers with a key role in cancer biology have also been investigated in order to identify subpopulations of CTCs with a higher potential for aggressiveness [35]. Epithelial growth factor receptor (EGFR) is expressed in different cell types except hematopoietic cells and is commonly used as a therapeutic target in CRC. As more and more anti-EGFR resistance is identified, it may become important to detect CTC modification in terms of its expression of EGFR during anti-EGFR treatment [41]. Similarly, as *TYMS* polymorphisms seem predict response to 5FU–based chemotherapy [91], which is the main chemotherapy used as a neoadjuvant setting before rectal surgery, and also the ultraviolet excision repair protein, RAD23 homolog B (RAD23B), which potentially be “induced by the genetic damage introduced by radiotherapy” [92] before rectal surgery, both monitoring of thymidylate synthase (TYMS) and excision repair protein, RAD23 homolog B (RAD23B), can be used to predict resistance to chemotherapy/radiotherapy used in rectal cancers [69].

### 5.3. The Future

Our updated review confirms the potential of CTC biomarker detection as a prognostic tool, in keeping with the findings of a previous meta-analysis [93]. Molecular characterization at both cellular and genomic levels may be of utility in the prognosis for patients with CRC, whatever the initial stage (metastatic or not). Furthermore, where CTCs remain present despite chemotherapy treatment, this may be used to indicate failure of therapy and predict survival. CTCs remain of great interest to clinicians who look after patients with CRC due to their potential for utilization in clinical practice. They might give a “snapshot” of the disease within time and space, and enable the individualised therapy for patients. This may be possible by identifying patients at risk, facilitate more accurate cancer surveillance and potentially the adaptation of treatments. One focus for the future application of such a test would be the search for the most sensitive and specific (and cost-effective) method for use in routine daily clinical practice.

There were a number of articles excluded because they focused on other liquid biopsy techniques. However, they demonstrate the breadth of interest in the search for applicable circulating prognostic biomarkers for CRC. Some include the “OMICS” (circulating tumoural DNA, miRNA, circulating protein), the microenvironment (tumour-derived exosomes, circulating immune cells with tumour associated macrophage) and neo-vascularisation (circulating endothelial cell, VEGF [Vascular Endothelial Growth Factor], EPC [endothelial progenitor cells], CEC [Circulating endothelial cells]) [94]. Interestingly, some of these other biomarkers made CTCs less prevalent. For example, DNA mutation analysis is now often assessed on circulating tumour DNA rather than on CTC [95].

Several interesting avenues require further evaluation concerning the detection and analysis of CTCs. There may be some role in early diagnosis through screening [96]. CTCs may also be helpful in identifying cancer primary site [97] in cases where there is diagnostic uncertainty. Further investigations regarding tumour biology and the metastatic process are in development, focusing on tumour microenvironment, the epithelial to mesenchymal transition, circulating cancer cell stemness, and others [98,99]. Furthermore, when CTC are isolated without being destroyed, CTC-derived xenografts could lead to patient specific drug screening [100,101,102].

## 6. Conclusions

We identified 77 studies that reported data regarding the prognostic utility of CTCs for patients with CRC. CTCs seem to have a high potential for use as a prognostic biomarker in CRC in both quantitative and qualitative terms. Ongoing investigations are required to evaluate the role of CTC analysis in routine clinical practice.

## Figures and Tables

**Table 1:** Exclusion criteria.

Exclusion Criteria	Number of Articles
Other elements than CTCs: in liquid biopsy, non-circulating, microenvironment	163
Not CRC	22
Review or case report	74
CTCs research without prognostic angle	65
Other (languages, …)	20
TOTAL	322

**Table 2:** Number of studies according to their marker type and results.

	Significant Difference	No Significant Difference	Total
**Quantity**			60
CellSearch	11	3	14
RT-QPCR	32	5	37
Other	9	0	9
**Quality**	9	0	9
**Quantity and Quality**	7	1	8

**Table 3:** Summary of quantitative included studies, separated into categories according to technique.

Study	Technique	Study Type	Marker of Prognosis Assessed	Number of Patients	Control Group	CRC Stage and Mutational Status	Results
Matsusaka et al., 2011 [5]	CellSearch	Prospective	CTC count at baseline (B1), and at 2 (B3), and 8–12 weeks (B4) after initiation of oxaliplatin-based chemotherapy (folfox4 with or without bevacizumab).	64	No	M + CRC	Patients with ≥ 3 CTC at B1, at B3, and at B4 had a shorter median PFS and median OS than those with <3 CTC.
Coumans et al., 2012 [6]	CellSearch	Prospective	CTC count before beginning (B1) of a new line of therapy and at first follow-up (B3).	428	Yes	M + CRC	For each 10-fold CTC raise, survival of patients with positive CTC count is reduced by 6.6 months.
Aggarwal et al., 2013 [7]	CellSearch	Prospective	CTC count at baseline (B1) and at 1–20 weeks (B3 et B4) after initiation of new therapy.	217	No	M + CRC with a baseline CEA value ≥ 25 ng/mL,	Patients with CTCs < 3 at B1 had longer survival than those with high CTCs. In a multivariate analysis, B1 CTCs status independently predicted survival.
Otsuka et al., 2013 [8]	CellSearch	Prospective	CTC count during chemotherapy (B2).	14	No	Stage III and IV M + CRC	They analysed the relationship between CTC count and therapeutic outcome. A non-significant negative correlation was observed.
Sastre et al., 2013 [9]	CellSearch	Prospective	CTC number at baseline (B1) and after cycle 3 of chemotherapy and bevacizumab (B3) and after disease progression (B5).	158	No	M + CCR with determined KRAS status	Patients with < 3 CTC per 7.5 mL blood at B1 and KRAS wild type tumours had a significant better median PFS and OS than patients with ≥ 3 CTCs and KRAS mutated tumours. This was confirmed by multivariate analyses.
Bork et al., 2015 [10]	CellSearch	Prospective	CTC count preoperatively (A1) and on postoperative days 3 and 7 (A3, A4).	287	No	CRC patients in 2 groups: complete patient group and the non-metastatic cohort (Stage I, II, and III).	Patients with ≥1 CTC per 7.5 mL had a significantly worse OS in the non-metastatic group, as well as in the complete cohort. This strong prognostic factor for both groups was confirm by multivariate analysis.
Krebs et al., 2015 [11]	CellSearch	Prospective	Baseline CTCs (B1) count allowing stratifying patients into low (<3) and high (≥3) CTC groups. Patients undergoing a 4-drug regimen called eSCOUT (irinotecan, oxaliplatin, and tegafur-uracil with leucovorin and cetuximab)	48	No	Previously untreated KRAS wild-type Advanced CRC (inoperable locally advanced or M+ CRC)	Compared with CRC undergoing CAIRO2 trial (3drug regimen). For patients with CTC < 3, no differences were found between the median OS in the eSCOUT trial and in the CAIRO2 trial. For patients with CTC > 3, median OS was significantly improved in the eSCOUT group.
Sotelo et al., 2015 [12]	CellSearch	Prospective	CTC count after primary tumour resection (A3) and before the start of adjuvant therapy (A4).	519	No	Stage III CRC	No significant relationship was found between CTC count and prognosis.
van Dalum et al., 2015 [13]	CellSearch	Prospective	CTC count before surgery (A1) and at several time-points during follow up (A3, A4).	183	Yes	Newly diagnosed (stage I, II and III) M + CRC scheduled for surgery	Patients with CTC at A1 had a significant decrease in RFS, and CCRS. When using a multivariate analysis, a significant correlation was found between A1 CTC presence and RFS. CTC detection during follow up was not significantly associated with RFS.
Connor et al., 2016 [14]	CellSearch	Prospective	CTC count in peripheral (PV) and hepatic vein (HV) at the time of the first liver operation (A2).	63	No	M + CRC with liver metastasis undergoing CRLM resection	Patients with HV CTCs > 3 had a significantly shorter DFS and OS. No significant correlation was found between PV CTCs and survival.
Rothé et al., 2019 [15]	CellSearch	Prospective	CTC count before therapy and after one course of preoperative FOLFOX.	36 patients	No	Stage III colon cancer undergoing 1 course of neoadjuvant FOLFOX treatment	CTC detection rate at baseline was low to continue inclusion in this sub-study.
Aranda et al., 2020 [16]	CellSearch	Prospective	CTC count at baseline (B1) before receiving either FOLFOXIRI + bevacizumab or FOLFOX + bevacizumab every two weeks.	349	No	M + CRC previously untreated, unresectable with ≥ 3 CTC/7.5 mL blood at baseline (B1)	For M+ patients with CTC≥3, median PFS was significantly higher with FOLFOXIRI bevacizumab than with FOLFOX-bevacizumab, although with a higher rate of serious toxicities.
Camera et al., 2020 [17]	CellSearch	Prospective	CTC count at day 1 and day 15 (B1). No patient received any cancer treatment between day 1 and 15.	55	No	Unresectable advanced CRCs that were refractory or intolerant to standard treatments.	Patients with high CTC count at B1 had a significant worse OS.
Bessa et al., 2001 [18]	CEA mRNA RT-PCR	Prospective	CTC presence before surgery (A1).	95	No	CRC (6 Stage I, 32 Stage II, 37 Stage III, 20 stage IV)	For the whole cohort or for the 68 patients undergoing curative surgery, no significant correlation was found between DFS or OS and CTC presence.
Sadahiro et al., 2001 [19]	CEA mRNA RT-PCR	NIF	CTC in total and peripheral blood during surgery (A1).	121	Yes	20 Stage I, 48 stage II, 34 stage III 18 stage IV All with curative resection	No significant correlation was found between positive rate and level of CEA mRNA and prognostic factors.
Guller et al., 2002 [20]	CEA and CK2O mRNA RT-PCR	Prospective	Tumour cells presence in peritoneal lavage, peripheral blood before and after surgery (A1, A3). In mesenteric blood, during surgery (A2).	39	No	7 Stage I, 14 stage II, 18 stage III All with curative resection	There was a significant correlation between dichotomous (CK20, CEA), qPCR covariate and DFS or OS.
Ito et al., 2002 [21]	CEA mRNA RT-PCR	NIF	CTC presence immediately before (A1) and after surgery (A3).	99	Yes	5 stage 0, 19 stage I, 27 stage II, 48 stage III All with curative resection	Patients with positive CEA mRNA in post-operative blood had a significantly shorter DFS than cases with negative CEA mRNA.
Bessa et al., 2003 [22]	CEA mRNA RT-PCR	Prospective	CTC presence 24 h after surgery (A3).	66	No	14 stage I, 28 stage II, 24 stage III All with curative resection	No significant correlation was found between CTCs presence and recurrence, OS, or CCRS.
Sadahiro et al., 2005 [23]	CEA mRNA RT-PCR	NIF	CTC presence during surgery (A2) in portal and peripheral blood.	100	No	20 stage I, 47 stage II, 32 stage III All with curative resection	Patients with CEA mRNA-positive portal blood had a lower 4-year recurrence rate than patients with CEA mRNA-negative portal blood. There was no significative correlation between DFS and CEA mRNA positivity.
Katsumata et al., 2006 [24]	CK20 mRNA RT-PCR	NIF	CTC presence during surgery (A2).	57	Yes	Dukes’ stage A, 7 cases Stage B, 26 cases Stage C, 14 cases Stage D, 10 cases All with curative resection	CK20(+) patients had a significant lower 5-year survival rate than CK20(-) patients.
Allen-Mersh et al., 2007 [25]	CEA and CK2O mRNA RT-PCR	NIF	CTC presence 24h before surgery (A1), and 24h (A3) and 1 week (A4) after surgery.	199	Yes	Dukes’ stage A, 30 cases Stage B, 69 cases Stage C, 47 cases One with no Dukes’ classification All with curative resection	Patients with A3 positive blood sample for either CEA or CK20 or both had a significantly lower DFS.
Friederichs et al., 2007 [26]	CK20 mRNA RT-PCR and density centrifugation	NIF	CTC presence at time of primary staging (A1or B1).	37	No	11 stages I, 9 stages II, 9 stages III 8 stages IV	No significant correlation was found between OS and CK20-positive cells presence. Only a trend toward better survival for CK20 (-) negative patients was found.
Sadahiro et al., 2007 [27]	CEA mRNA RT-PCR	NIF	CTC presence between 7 and 10 days after surgery (A3).	200	No	55 stages I, 86 stages II, 55 stages III all with curative surgery	No correlation was found between detection of CEA mRNA and clinicopathological findings. Patients with positive CEA mRNA showed significantly poorer DFS and OS than the negative patients.
Koyanagi et al., 2008 [28]	4 marker mRNA RT-PCR	Prospective	CTC presence.	34	Yes	CRC patients with sentinel lymph node 15% T1 tumours, 18% T2 and 67% T3	There was a significant correlation between RT-qPCR analysis of blood for CTCs and OS.
Yie et al., 2008 [29]	Survivin mRNA RT-PCR and ELISA	NIF	CTC presence at base line (A1 or B1).	86	Yes	21 cases stage I, 34 cases stage II, 22 cases stage III 9 cases stage IV	There was a significant correlation between survivin-expressing CTCs presence at baseline (A1 or B1) and the degree of tumour penetration, nodal status, disease stages and relapse rate.
Tsouma et al., 2010 [30]	CEA, CK20 and EGFR mRNA RT-PCR	NIF	CTC presence preoperatively (A1).	88	Yes	Astler–Coller classification: 26 cases stage B, 27 cases stage C 31 cases stage D All with curative surgery	6 groups of CRC patients were created: group 0: no marker expression; group 1: positive only for CEA; group 2: positive for CEA and CK20; group 3: positive for all three markers; group 4: patients of groups 1 and 2 and group 5: patients of groups 2 and 3. There was a significant correlation between OS and the different CRC patient groups.
Iinuma et al., 2011 [31]	CEA, CK19, CK20 and/or CD133 RT-PCR	Retrospective training set and a Prospective validation set	CTC presence at the pre surgery stage and before chemotherapy begun (A1 or B1).	735	No	Dukes’ stage A, 169 cases Stage B, 319 cases Stage C, 247 cases	Patients with positive CEA/CK/CD 133 had a significantly worse OS and DFS than those of patients who were negative.
Vardakis et al., 2011 [32]	CEACAM5 mRNA RT-PCR	NIF	CTC presence after surgery and before adjuvant systemic therapy (A3 or A4).	265	Yes	105 stage II 160 stage III with curative surgery	There was a significant correlation between detection of CEACAM5m RNA-positive cells and relapse rate, DFS, death rate and lower median OS.
de Albuquerque et al., 2012 [33]	KRT19, MUC1, EpCAM, CEACAM5 and BIRC5 RT-PCR	NIF	CTC presence at baseline (B1).	60	No	CRC initiating any first or second-line systemic therapy. 4 cases stage II, 8 cases stage III 48 stages IV	Patients with CTC positivity (at least one of the marker genes was positive) at B1 had a significant shorter median PFS than patients with no CTCs (all marker genes were negative).
Liu et al., 2012 [34]	CEA and CK2O mRNA RT-PCR	NIF	CTC presence on the day of initiation of adjuvant chemotherapy (stage II and III)(A4), or on the first day of chemotherapy after diagnosis (M+) (B1).	46	Yes	5 cases stage I, 8 cases stage II, 14 cases stage III 19 cases stage IV	Stage IV patients had a higher levels of CEA mRNA, CK20 mRNA than patients at stages I– III. There was a significant correlation between peripheral blood CEA mRNA levels and overall survival.
Pilati et al., 2012 [35]	CEA, CK19, CK20, VEGF, EGFR, Survivin and CD133 RT-PCR	Retrospective	CTC presence immediately before surgery (A1).	50	Yes	M + CRC with liver metastasis undergoing CRLM resection	There was a significant correlation between expression levels of CD133 and patient survival.
Shimada et al., 2012 [36]	CEA, CK19, CK20 and/or CD133 RT-PCR	NIF	CTC presence from the mesenteric vein during surgery (A2).	197	Yes	111 CRC with Dukes’ stage B 86 cases with stage C all with curative surgery	There was a significant correlation between CEA/CK/CD 133 expression and Dukesʹ stage. Patients with Dukesʹ stage B and C, with CEA/CK/CD 133 positive in the tumour drainage blood had a significantly worst DFS and OS than negative patients.
Yokobori et al., 2013 [37]	PLS3 RT-PCR	NIF	CTC presence before surgery (A1)	711	Yes	Dukes’ Stage A, 85 cases Stage B, 309 cases Stage C, 234 cases Stage D, 83 cases	There was a significant correlation between PLS3-positive CTC and prognosis in the validation set. The association was particularly strong in patients with Dukes B and Dukes C stage.
Barbazan et al., 2014 [38]	GAPDH, VIL1, CLU, TIMP1, LOXL3 and ZEB2 mRNA RT-PCR	NIF	CTC presence at baseline (B1) and at 4 (B3) and 16 weeks (B4) after treatment onset.	50	Yes	M + CRC treated by fluoropyrimidine alone or in combination with oxaliplatin/irinotecan and biological targeted therapies (bevacizumab, cetuximab)	They classified patients with at least four of the six-gene panel markers below cut-offs, as low-CTC. Moreover, those with three or more markers above cut-offs in the high-CTC group. Patients with high B1 CTC had a significant lower median PFS and OS than patients with low CTC markers.
Hinz et al., 2015 [39]	CK20 mRNA RT-PCR	Prospective	CTC presence at the time of surgery.	267	No	Rectal locally advanced tumours that were treated with chemoradiation followed by surgery	Non-responders had a significantly higher CTC count than responders. There was a significant correlation between OS and responder status.
Katoh et al., 2015 [40]	CD44 variant exon9 (CD44v9) mRNA RT-PCR	NIF	CTC presence.	150	Yes	Stage I, 24 cases Stage II, 35 cases Stage III 55 cases Stage IV, 36 cases of sporadic CRC	In patients with stage III disease, patients with negative CD44v9 expression had a significantly higher 5-year survival rate than patients with positive expression. In patients with stage IV unresectable cancer, CD44v9-negative expression patients had a higher 2-year survival rate than patients with positive expression.
Musella et al., 2015 [41]	CEA, EGFR and EpCAM mRNA RT-PCR	Prospective	CTC detection at baseline (B1), during treatment (B2), at early (2–4weeks) and at later time (8–10 weeks).	38	No	M+ chemorefractory, RAS-BRAF wild-type CRC receiving third-line therapy with cetuximab or panitumumab	Samples were CTC-positive if at least one of the 3 genes was above the threshold. There was a significant correlation between CTC status, CTC status changes during treatment and tumour response. Patients with early CTC(+) status had a significantly shorter PFS and OS than CTC(-)ones.
Ning et al., 2015 [42]	Survivin and CK20 mRNA RT-PCR	Retrospective	CTC presence at baseline (A1 or B1).	88	Yes	M + CRC	Patients with high CTC-CK20 expression or high CTC-survivin expression had a significantly shortened median OS than those with low expression of either marker.
Kust et al., 2016 [43]	CK20 mRNA RT-PCR	NIF	CTC presence on the day of the surgery (A1) and 5 to 7 (A3) days after surgery.	95	Yes	Stage I, 22 cases Stage II, 30 cases Stage III, 30 cases Stage IV, 13 cases	Patients positive for CK20 postoperatively (A1) had significant shorter PFS and OS than CK20 negative patients.
Hinz et al., 2017 [44]	CK20 mRNA RT-PCR	Prospective	CTC presence at time of surgery (A1).	299	Yes	CRC with curative surgery: Stage I, 87 cases Stage II, 94 cases stage III, 80 cases Stage IV, 38 cases	Patients with detectable CTC had a significant lower 5-year OS rate than patients without detectable CTC.
Insua et al., 2017 [45]	GAPDH, VIL1, CLU, TIMP1, TLN1, LOXL3 and ZEB2 mRNA RT-PCR	Prospective	CTC presence before (B1) and at 4 and 16 weeks (B2) after treatment onset.	94	No	M + CRC treated bay first-line antineoplastic treatment	Patients where in the high/low CTCs groups, when at least four markers were above/below the individual cut-offs. There was a significant correlation between all CTC markers that presented an expression above the cut-off (high CTC) and shorter OS and PFS rates, both when analysed at B1. Patients with increased expression of CTC markers during treatment had a shorter PFS and OS times than patients presenting decreased expression.
Ning et al., 2018 [46]	PI3Ka, Akt-2, Twist2 and ALDH1mRNA RT-PCR	NIF	CTC presence before treatment (B1).	78	Yes	M + CRC	Patients with positive CTC Akt-2 expression had a significantly shorter median PFS than those without. Patient with positive CTC ALDH1 expression had a significantly shorter OS than negative patient.
Shou et al., 2019 [47]	CEA, CK19, EpCAM, MUC1, EGFR and c-Met RT-PCR	NIF	CTC presence at diagnosis (B1).	50	Yes	38 cases Stage III 12 cases stage IV relapsed	There was a significant correlation between CTC numbers measured by six-gene assay and 2-year PFS.
Uen et al., 2007 [48]	Other (mRNA markers detected by colorimetric membrane-array)	Prospective	CTC detection at least 1 week after surgery (A3).	194	Yes	Stage II CRC with curative surgery	There was a significant univariate correlation between postoperative relapse and the expression of all 4 mRNA markers. For stage II patient, there was a significant correlation between the expression of all 4 mRNA markers and poorer relapse-free survival rates.
Lu et al., 2011 [49]	Other (mRNA markers detected by membrane-array)	Retrospective	CTC detection at 1 (A3) and 4 weeks (A4) after surgery.	141	No	Stage II and III CRC with curative surgery	CTCs presence was positive if patients were overexpressing all 4 molecular markers in peripheral blood samples at A3 and A4. There was a significant correlation between persistent postoperative CTCs and early postoperative (within one year) relapse, poorer DFS and OS.
Lu et al., 2013 [50]	Other (mRNA markers detected by membrane-array)	Retrospective	CTC detection at 1 (B3) and 4 weeks (B4) after completion of chemotherapy.	90	No	Stage III CRC with curative resection followed by 12 cycles of mFOLFOX	Patients had persistent presence of CTC if they over expressed all 4 molecular markers at both 1 (B3) and 4 weeks (B4) after chemotherapy. There was a significant correlation between persistent presence of post-chemotherap eutic CTCs and post-chemotherapeutic relapse, reduced DFS and OS.
Divella et al., 2014 [51]	Other	Prospective	CTC detection on the day of diagnosis.	103	Yes	M + CRC, (synchronous or metachronous hepatic and pulmonary metastases)	They detected 2 different populations of CTCs: single cells or clusters. There was a significant correlation between the presence of clustered CTCs and reduced OS.
Chang et al., 2016 [52]	Other (multigene biomarker detected by membrane array)	Prospective	CTC detection during follow-up surgery (A3).	298	No	82 cases Stage I, 102 cases Stage II 114 cases Stage III	There was a significant correlation between post-op (A3) relapse, lower DFS, OS, and positive biochip results.
Wu et al., 2017 [53]	Other (SE-IFISH)	Prospective	CTC detection, 1 day before (A1), 1 week after (A3) and 3-month after (A4).	44	Yes	28 cases stage I and II 16 cases of stage III–IV	No significant correlation was found between survival and A1 CTC count. At A4, CTC < 2 patients had a significantly longer PFS than those with CTC ≥ 2. There was a significant correlation between decreased CTC number (compared with preoperative CTC number) and longer PFS.
Chou et al., 2018 [54]	Other (flow cytometry)	Prospective	CTC detection at baseline (B1).	55	No	M + CRC undergoing palliative chemotherapy	Patients with CTC number ≤ 30/mL had significant lower median OS and PFS than patients with CTC number > 30/mL. There was a significant c on between median OS or PFS and prognostic group (including CTC status).
Yang et al., 2018 [55]	Other (ISET)	Prospective	CTC detection before (A1) and after surgery (A3).	138	No	13 cases Stage I, 67 cases stage II, 58 cases stage III	With a multivariate analysis, there was a significant correlation between post-op, but not pre-op CTCs+ and shorter 3-year RFS rate.

Abbreviations: carcinoembryonic antigen (CEA), colon cancer related survival (CCRS), guanylyl cyclase C (GCC), cytokeratin 19 (CK19), cytokeratin 20 (CK20), colorectal liver metastasis (CRLM), confidence interval (CI), disease-free survival (DFS), epidermal growth factor receptor (EGFR), hazard ratio (HR), isolation by Size of Epithelial Tumour Cells (ISET), metastatic colorectal cancer (M + CRC), non-metastatic colorectal cancer (M − CRC), messenger ribonucleic acid (mRNA), no information found (NIF), overall survival (OS), progression-free survival (PFS), Plastin3 (PLS3), real-time quantitative polymerase chain reaction (qPCR), recurrence-free survival (RFS), relative risk (RR), reverse transcriptase polymerase chain reaction (RT-PCR), real-time reverse transcriptase-polymerase chain reaction (RT-qPCR), subtraction enrichment and immunostaining fluorescence in situ hybridization (SE-iFISH), time-to-therapeutic failure (TTF), vascular endothelial growth factor (VEGF).

**Table 4:** Summary of qualitative included studies.

Study	Technique	Study Type	Marker of Prognosis Assessed	Number of Patients	Control Group?	CRC Stage and Mutational Status before Any Treatment	Results
Iinuma et al., 2000 [56]	MACS and nested MASA on p53 and KRAS genes	NIF	CTC detection from drainage veins during surgery (A2).	23	No	Dukes’ stage A, 5 cases Stage B, 4 cases Stage C, 9 cases Stage D, 5 cases	There was no significant correlation between positive rate of the mutated gene in the blood and tumour stage. Patient with p53 and/or K-RAS gene mutation-positive findings had significantly shorter OS than patients testing negative.
Gazzaniga et al., 2010 [57]	ALDH1, CD44, CD133, MRP5 and survivin expression	NIF	CTCs detection at the start of chemotherapy (B1).	40	No	M + CRC patients treated with oxaliplatin (L-OHP) and 5-fluorouracil (5-FU) based regimens	There was no significant correlation between the expression of either CD44 or CD133 in CTCs and the outcome of patients. There was a significant correlation between the presence of CTCs positive for the expression of ALDH1, survivin and MRP5 and shorter PFS.
Tseng et al., 2015 [58]	DC133 and ESA expression by flow cytometry	Prospective	CTC detection in peripheral blood before the surgery (A1) and from mesenteric venous blood (MSV) during surgery (A2).	135	No	88 cases of stage I and II 47 cases of stage III CRC, all undergoing curative surgery	With a multivariate Cox analysis, they found a significant correlation between analysis of CD133+/ESA + cells in MVB and both DFS and OS.
Abdallah et al., 2016 [59]	ISET and immunocytochemical expression of MRP1, MRP4 and ERCC1	Prospective	CTC detection before the beginning of chemotherapy (B1).	34	Yes	M + CRC who were initiating a new line of irinotecan/oxaliplatin/5-FU	For patients treated with irinotecan-based chemotherapy, patients with MRP1 positive CTCs showed a worse PFS than patients with MRP1 negative CTCs.
Barbazan et al., 2016 [60]	GEFs expression of EpCAM-negative isolated cells	NIF	NIF	51	Yes	M + CRC	There was a significant correlation between a shorter median time to progression, a lower PFS and a high GEF expression.
Satelli et al., 2016 [61]	nuclear, membrane and cytoplasm PDL1 expression using confocal microscopy	Retrospective	CTC detection at random time during routine evaluation (B3).	62	Yes	M + CRC refractory to 5-FU treatment, undergoing palliative chemotherapy	There was no significant correlation between CTC detection alone and progression-free or OS. However, there was a significant correlation between nuclear PD-L1 (nPD-L1) expression and short survival.
Fang et al., 2017 [62]	CD133, CD54 and CD44 expression by flow cytometry	Prospective	CTC detection before surgery (A1) or chemotherapy (B1).	152	No	M + CRC with liver metastasis	In a multivariate analysis, patients with high expression of CD133 + CD44 + CD54 + cellular subpopulations had a worse survival than those patients with low expression. However, the survival of patients who had resection for primary tumour accompanied by surgical treatment for metastasis was not correlated with the expression of CD133 + CD44 + CD54 + subpopulation of CTCs.

Abbreviations: disease-free survival (DFS), epithelial-specific antigen (ESA), guanine nucleotide exchange factors (GEFs), magnetic-activated cell separation or sorting (MACS), mutant allele-specific amplification (MASA), no information found (NIF), overall survival (OS), Plastin-3 (PL S3).

**Table 5:** Summary of included studies that used both quantitative and qualitative markers.

Study	Technique	Study Type	Marker of Prognosis Assessed	Number of Patients	Control Group	CRC Stage and Mutation Status before Any Treatment	Results
Wong et al., 2009 [63]	CK20 expression by immunocytochemical staining	NIF	CTCs detection at baseline (A1 or B1) and on first follow up (A3 or B3).	132	Yes	101 cases of stage I to III 31 cases M+	There was a significant correlation between CK20 pCTC numbers and tumour node metastasis stage and lymph node status. Using the median CK20 pCTC numbers as the cut-off points, there was a significant correlation between recurrence, metastasis, survival and stratified groups of colorectal cancer patients.
Kuboki et al., 2013 [64]	EGFR expression	Prospective	CTC detection at baseline (B1).	63	No	KRAS wild type M + CRC receiving cetuximab as third line treatment	Patients with a high CTC count had a significantly lower OS than patients with a low number of CTCs. No significant correlation was found between PFS and high/low CTC status. There was no significant correlation between clinical outcome and EGFR status.
Abdallah et al., 2015 [65]	TYMS expression by immunochemical staining	Retrospective	CTC detection before chemotherapy (B1).	54	No	M + CRC starting a 5-FU based chemotherapy	No significant association was found between CTC TYMS staining and DP. There was a significant correlation between CTC count above 2 CTCs/mL and TYMS expression, correlating with worse prognosis.
Melero et al., 2016 [66]	EGFR expression by immunochemical staining	NIF	CTC detection at baseline (A1) and 6 weeks after surgery (A4).	14	No	M + CRC with peritoneal carcinomatosis and suitable for treatment with CRS and HIPEC	There was a significant association between the presence of CTCs and distant dissemination and PFS. No significant association was found between EGFR expression in CTCs at A1 and the presence of tumour dissemination.
Zhao et al., 2017 [67]	ISH	NIF	NIF	1203	Yes	213 cases stage I, 438 cases stage II, 249 cases stage III 303 cases stage IV	Based on the expression of epithelial and mesenchymal markers, three phenotypes were defined: epithelial, biphenotypic, and mesenchymal CTCs. There was a significant correlation between CTC numbers and clinical stage, lymph node metastasis and distant metastasis. Biphenotypic and mesenchymal, but not epithelial CTCs, were associated with the above clinical features.
Bahnassy et al., 2019 [68]	CK19, MUC1, CD44, C133, ALDH1 mRNA expression	Prospective	CTC detection on 2 peripheral blood sample.	63	Yes	M − CRC: 33 cases stages I and II, 30 cases stage III	In multivariate analysis, there was a significant correlation between positive CTCs for mRNA markers expression and shorter 5-y PFS and OS.
Troncarelli Flores et al., 2019 [69]	TYMS and RAD23 expression by immunochemical staining	Prospective	CTC detection before neoadjuvant chemoradiation (S1) and before (S2) surgery.	30	No	Locally advanced rectal cancer undergoing neoadjuvant chemoradiation followed by surgery	There was a significant correlation between CTC counts decreased between S1 and S2 in patients and pCR or partial response. Patients exhibiting pCR had negative TYMS and RAD23B CTCs. There was an association between, RAD23B expression and non-responder status at S1 and S2.

Abbreviations: cytoreductive surgery (CRS), disease-free survival (DFS), disease progression (DP), hyperthermic intraperitoneal chemotherapy (HIPEC), in situ hybridization (ISH), non-metastatic colorectal cancer (M − CRC), no information found (NIF), overall survival (OS), pathologic complete response (pCR), thymidylate synthase (TYMS).
