# Supplementary figures and images for: Effect of Quercetin on ABCC6 Transporter: Implication in HepG2 Migration
Source: Int J Mol Sci. 2021 Mar 26;22(7):3437. doi: 10.3390/ijms22073437 (PMC8068395; doi:10.3390/ijms22073437)

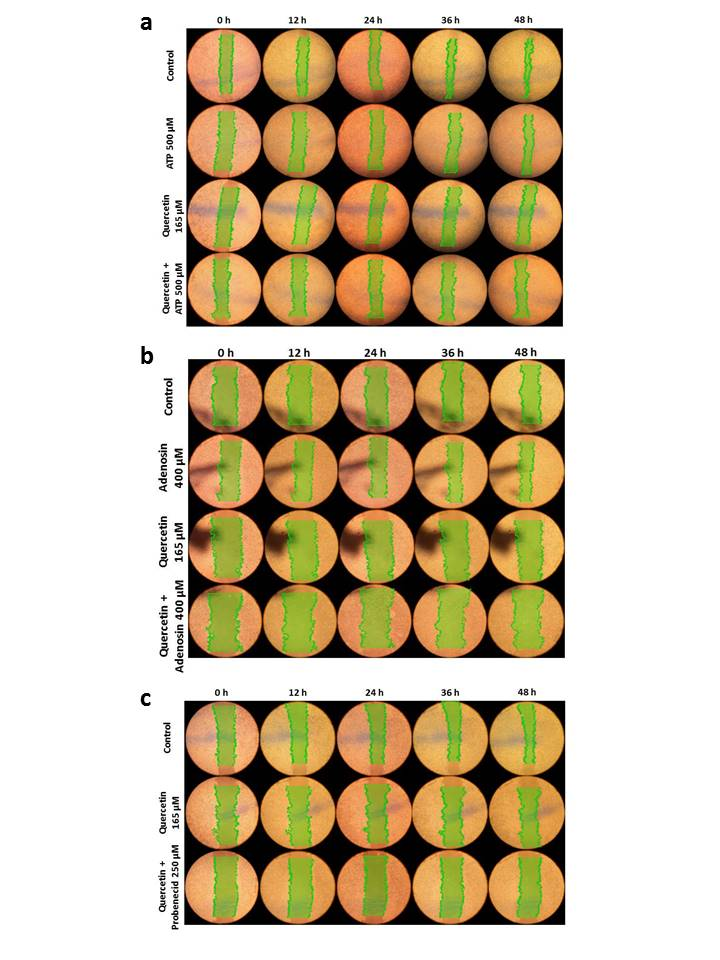

Supplement: Supplementary file 1 [file ijms-22-03437-s001.zip › ijms-1159040-SI.tif]
